# Successful conservative treatment of type A aortic intramural hematoma with typical CT evolution: a case report

**DOI:** 10.3389/fcvm.2025.1648753

**Published:** 2025-10-10

**Authors:** Jinlin Wu, Jiayu Song, Jie Liu

**Affiliations:** Department of Cardiac Surgery, Guangdong Provincial People’s Hospital (Guangdong Academy of Medical Sciences), Southern Medical University, Guangzhou, China

**Keywords:** acute aortic syndrome, aortic intramural hematoma, conservative treatment, case report, aorta, ascending aorta

## Abstract

Type A aortic intramural hematoma (IMH) is a variant of acute aortic syndrome, with emergency surgery traditionally considered the treatment of choice. We report a case of a 69-year-old male patient who presented with chest pain. Computed tomography angiography (CTA) revealed type A aortic intramural hematoma. Given the absence of high-risk progression factors, conservative management with close surveillance was selected. During treatment, periaortic hematoma developed in the descending aorta, but the patient's symptoms significantly improved. Follow-up CTA at 3 months demonstrated complete resolution of the intramural hematoma, and the patient remained in good condition at 2.5 years of follow-up. This case demonstrates that conservative treatment may be a viable option for highly selected type A IMH patients without high-risk progression factors, but should only be considered with rigorous patient selection criteria and intensive monitoring protocols in experienced centers.

## Introduction

Acute aortic syndrome (AAS) encompasses a group of aortic wall diseases with high mortality and morbidity rates (1). The mechanism of IMH likely involves spontaneous rupture of the vasa vasorum within the aortic media, leading to intramural bleeding without intimal tear.

Type A IMH represents a cardiothoracic emergency requiring surgical evaluation regardless of patient symptoms and clinical stability, due to increased risk of progression to aortic dissection or rupture. Based on findings from the International Registry of Acute Aortic Dissection (IRAD), conservative treatment is not recommended for type A IMH due to mortality rates of approximately 40% (2). However, controversy remains regarding the optimal treatment strategy for type A IMH, particularly considering geographical and ethnic variations. Asian studies demonstrate lower mortality rates with conservative treatment, while Western reports show higher mortality with conservative management. The 2022 ACC/AHA Guidelines recommend immediate open surgery for uncomplicated IMH (Class I recommendation) (3). Similarly, the 2024 EACTS/STS Guidelines state that emergency surgery is recommended for patients with acute type A IMH with complications or high-risk features (Class I recommendation) (4). However, these same guidelines acknowledge that optimal medical therapy and serial imaging may be considered in patients with type A IMH in the absence of high-risk features. This reflects the ongoing controversy regarding optimal treatment strategy for type A IMH.

This report presents a case of type A IMH treated conservatively with follow-up studies demonstrating disease improvement and favorable outcomes.

## Case description

A 69-year-old male patient (height 165 cm, weight 55 kg) presented to the emergency department on December 14, 2022, with chest pain. The chest pain was persistent, not relieved by rest, and gradually worsened over time. The patient had no history of hypertension or diabetes mellitus but was a smoker. Physical examination revealed that the patient was alert and conscious with stable vital signs: blood pressure 140/80 mmHg, heart rate 85 beats per minute, respiratory rate 16 breaths per minute, and temperature 36.8°C. Cardiopulmonary and abdominal examinations were unremarkable, and peripheral pulses were symmetric.

Electrocardiography showed sinus rhythm without significant abnormalities. Chest CTA revealed type A aortic intramural hematoma extending from the ascending aorta to the descending aorta. The ascending aortic length was 108 mm, maximum aortic diameter was 42 mm, and hematoma thickness was 7 mm, without associated ulcer-like projection (ULP). The aortic valve was tricuspid without significant aortic regurgitation.

Following multidisciplinary discussion, considering the absence of high-risk progression factors (aortic diameter <50 mm, hematoma thickness <10 mm, no ULP) and the patient's advanced age, conservative treatment was selected. The patient received strict pain control, heart rate and blood pressure control with target systolic blood pressure of 100–120 mmHg and heart rate of 60–80 bpm. Intravenous medications were used for the first three days, then gradually transitioned to oral medications for ongoing control. Strict bed rest was mandatory during hospitalization. The patient was closely monitored during the entire hospital stay. CTA was recommended at 1 weeks, 2 weeks, 1 month, 3 months, 6 months, and then annually.

**Figure 1:**
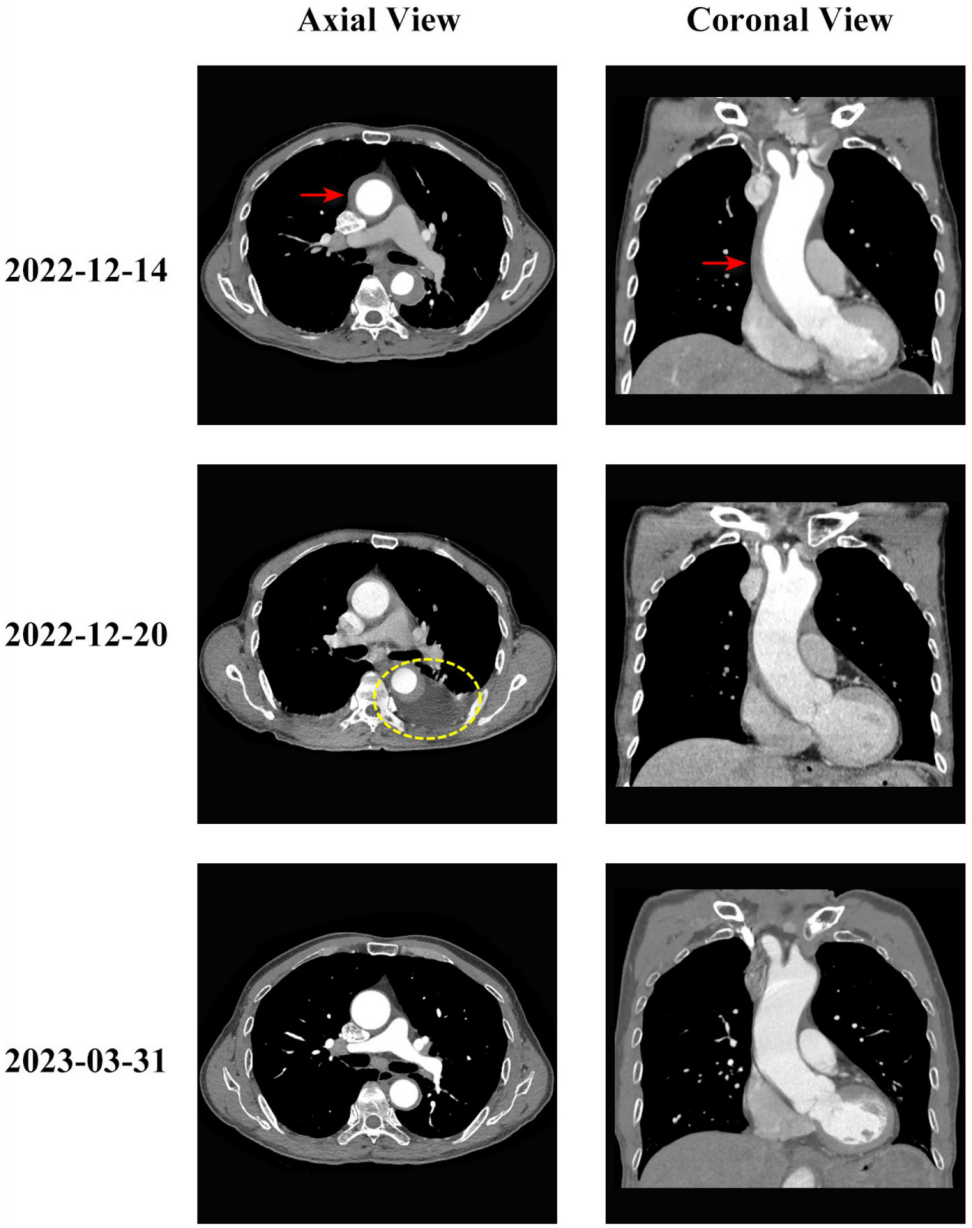
Evolution of type A aortic intramural hematoma during conservative treatment. Serial computed tomography angiography (CTA) images showing the evolution of type A aortic intramural hematoma in axial (left column) and coronal (right column) views. **Top row** (2022-12-14): Initial presentation showing type A intramural hematoma involving the ascending aorta (red arrows indicate intramural hematoma location) with thickening of the aortic wall (hematoma thickness 7 mm). The ascending aortic diameter measured 42 mm. **Middle row** (2022-12-20): One weeks after conservative treatment initiation, showing resolution of the ascending aortic intramural hematoma but development of periaortic hematoma around the descending aorta (yellow circle indicates periaortic hematoma). **Bottom row** (2023-03-31): Three months follow-up demonstrating complete resolution of both the intramural hematoma and periaortic hematoma, with restoration of normal aortic wall appearance. The patient remained asymptomatic throughout the follow-up period.

Follow-up CTA one week after treatment showed resolution of the ascending aortic intramural hematoma, but periaortic hematoma was noted in the descending aorta. The patient's chest pain symptoms significantly improved, and vital signs remained stable. Follow-up aortic CTA at 3 months post-discharge demonstrated complete resolution of the ascending aortic hematoma and complete disappearance of the periaortic hematoma. The patient returned to normal activities, and clinical follow-up at 2.5 years showed continued good clinical condition. The typical CT evolution process is shown in Figure 1.

## Discussion

### Controversy in type A aortic intramural hematoma treatment

Treatment strategies for type A IMH show significant geographical and ethnic variations. Asian centers, particularly studies from Japan and Korea, demonstrate that conservative treatment can achieve favorable outcomes for patients with hematoma thickness <11 mm and aortic diameter <50 mm, with in-hospital mortality rates <10% (5). In contrast, Western guidelines recommend aggressive surgical treatment, considering that type A IMH behaves similarly to aortic dissection, with conservative treatment mortality rates as high as 40% (1). These study differences likely arise from failure to strictly distinguish between simple and complicated type A hematomas in study inclusion criteria. Additionally, some hematomas may represent the false impression of thrombosed type A dissections. Vigilance regarding this heterogeneity is essential when interpreting results from different studies. Genetic variations and healing responses between ethnic populations might also influence natural history.

### Risk factors predicting hematoma progression

Identifying high-risk progression factors is crucial for selecting appropriate treatment strategies. Research indicates that a maximum diameter of the ascending aorta and aortic arch ≥50 mm and hematoma thickness ≥10 mm correlate with mortality in conservative treatment (6). Additionally, ULP, pericardial effusion, uncontrollable chest pain, and hypertension are important factors predicting progression. Our previous research also identified ascending aortic length as a strong risk factor for predicting progression (7).

This 69-year-old patient had no history of diabetes or coronary artery disease, no significant pericardial effusion or valvular disease. The ascending aortic diameter and hematoma thickness were 42 mm and 7 mm, respectively, and CTA revealed no ULP or intimal rupture. Due to the absence of high-risk factors for progression to aortic dissection, conservative treatment was selected. It is crucial to emphasize that the safety and efficacy of conservative management demonstrated in this single case should not be generalized to the broader population of type A IMH patients. This case represents a highly selected patient with specific favorable characteristics. In summary, key factors for successful conservative treatment in this case included: (1) strict blood pressure and heart rate control; (2) close clinical monitoring; (3) timely imaging follow-up; (4) absence of high-risk progression factors in the patient. The combination of these elements ensured treatment safety and effectiveness.

### Clinical significance of periaortic hematoma

The unique aspect of this case was the development of periaortic hematoma during conservative treatment. Mukherjee et al. (8) found in the International Registry of Acute Aortic Dissection that periaortic hematoma may be a sign of impending rupture and associated with adverse outcomes. However, these finding lacks confirmation from other centers, and its clinical significance in IMH patients remains unclear. In this case, despite the appearance of periaortic hematoma, the patient's symptoms significantly improved, and subsequent follow-up showed complete hematoma resolution, suggesting that in certain circumstances, periaortic hematoma may not necessarily predict adverse outcomes. This may relate to hematoma size, location, and the patient's overall clinical status. However, this highly unusual favorable outcome should not be interpreted as suggesting that periaortic hematoma is benign or that conservative management is appropriate when this finding develops. More evidence is warranted.

## Conclusion

This case report demonstrates that for type A aortic intramural hematoma patients without high-risk progression factors, conservative treatment is a viable therapeutic option under strict blood pressure and heart rate control with close clinical and imaging surveillance. This experience provides important reference for individualized treatment of type A IMH, but additional clinical studies are needed to validate the safety and effectiveness of conservative treatment.

## Data Availability

The original contributions presented in the study are included in the article/Supplementary Material, further inquiries can be directed to the corresponding author.
